# Applying quantitative bias analysis to estimate the plausible effects of selection bias in a cluster randomised controlled trial: secondary analysis of the Primary care Osteoarthritis Screening Trial (POST)

**DOI:** 10.1186/s13063-017-2329-1

**Published:** 2017-12-04

**Authors:** L. A. Barnett, M. Lewis, C. D. Mallen, G. Peat

**Affiliations:** Arthritis Research UK Primary Care Centre, Research Institute for Primary Care and Health Sciences, Keele University, Staffordshire, ST5 5BG UK

**Keywords:** Quantitative bias analysis, c-RCTs, Osteoarthritis

## Abstract

**Background:**

Selection bias is a concern when designing cluster randomised controlled trials (c-RCT). Despite addressing potential issues at the design stage, bias cannot always be eradicated from a trial design. The application of bias analysis presents an important step forward in evaluating whether trial findings are credible. The aim of this paper is to give an example of the technique to quantify potential selection bias in c-RCTs.

**Methods:**

This analysis uses data from the Primary care Osteoarthritis Screening Trial (POST). The primary aim of this trial was to test whether screening for anxiety and depression, and providing appropriate care for patients consulting their GP with osteoarthritis would improve clinical outcomes. Quantitative bias analysis is a seldom-used technique that can quantify types of bias present in studies. Due to lack of information on the selection probability, probabilistic bias analysis with a range of triangular distributions was also used, applied at all three follow-up time points; 3, 6, and 12 months post consultation. A simple bias analysis was also applied to the study.

**Results:**

Worse pain outcomes were observed among intervention participants than control participants (crude odds ratio at 3, 6, and 12 months: 1.30 (95% CI 1.01, 1.67), 1.39 (1.07, 1.80), and 1.17 (95% CI 0.90, 1.53), respectively). Probabilistic bias analysis suggested that the observed effect became statistically non-significant if the selection probability ratio was between 1.2 and 1.4. Selection probability ratios of > 1.8 were needed to mask a statistically significant benefit of the intervention.

**Conclusions:**

The use of probabilistic bias analysis in this c-RCT suggested that worse outcomes observed in the intervention arm could plausibly be attributed to selection bias. A very large degree of selection of bias was needed to mask a beneficial effect of intervention making this interpretation less plausible.

## Background

Cluster randomised controlled trials (c-RCT) are increasingly being used to evaluate the intended effects of complex interventions in primary care [1] and other health and social care settings [2]. The decision to choose cluster randomisation over individual patient randomisation is typically justified by the intervention being directed at the level of the cluster (practitioner, practice, hospital, etc.), or on the grounds of perceived risk of experimental contamination, or on cost, compliance, investigator cooperation, and other logistic considerations [3]. While c-RCT designs may offer a solution to these problems, and sometimes offer additional advantages (e.g. external validity), they also raise a number of specific methodological and ethical issues. Among these issues, vulnerability to selection bias is an important concern in c-RCT designs where individual participants are identified and recruited *after* randomisation, especially when the person identifying and recruiting participants is not blinded to allocation and the process of identification and/or recruitment is open to interpretation [4].

Prevention of bias is the ideal and a range of strategies have been proposed to minimise selection bias in the design and implementation phases of c-RCTs [5, 6]. However, in instances where these strategies are not, or cannot be, adopted, or where their success is not assured, it is important to carefully evaluate the potential role of selection bias in the interpretation of the trial findings. In the context of epidemiological studies, quantitative bias analysis (QBA) has been proposed as an approach to evaluating the role of bias in published research and an alternative to the traditional handling of this by qualitative judgements and educated guesses in the Discussion sections of articles [7–10]. Quantitative bias analysis involves quantifying the magnitude, direction, and effect of bias present in studies [11]. There are three types of bias that can be adjusted for; misclassification, unmeasured confounders, and selection bias which this paper will investigate. Selection bias occurs when the intervention and outcome are both conditioned on subject participation; a common problem in c-RCTs where subjects are not individually randomised. If all eligible participants do not get enrolled into the study and this is related to outcome and intervention, then any association found from the analyses conducted on the data with those who did contribute data will be different to any associations that would be found when using all eligible subjects [12]. The motivation for conducting a QBA is to adjust the estimate for the association between exposure/intervention and outcome for the presence of selection bias, induced by conditional participation.

To our knowledge, to date there has been little application of QBA to c-RCTs including the specific matter of how robust the findings are to difference in selection between intervention and control arms after randomisation. We conducted a small, non-systematic scoping review of Cochrane Library full-text papers published between 2013 and 2016 and reporting original findings c-RCTs available in the Cochrane Library. We chose Cochrane due to its broad scope of published trial papers. We identified a total of 35 trials; 13 commented on selection bias, of which four discussed its role on study findings, but none reported any formal quantitative evaluation of its role.

The aim of this paper was to apply quantitative bias techniques to a c-RCT whose design rendered it vulnerable to selection bias, in order to evaluate the extent to which different degrees of selection bias would modify the estimated effect of intervention and the conclusions drawn from it.

## Methods

### Data source

This QBA used data from the Primary care Osteoarthritis Screening Trial (POST) – a pragmatic, cluster randomised, parallel, two-arm trial in primary care in which 45 practices were block-randomised 1:1 to intervention or control using a balance algorithm based on list size, area deprivation and clinical commissioning group (CCG). When patients consulted for osteoarthritis during the study period, and an osteoarthritis diagnostic/symptom code was recorded by the general practitioner (GP) in their electronic record, a point-of-care electronic template was activated which was used to record screening data, prompt GPs to ask screening questions and identify those potentially eligible for inclusion. The intervention was point-of-care anxiety and depression screening and pain intensity assessment by the GP. The control was point-of-care pain intensity assessment by the GP, similarly prompted by an electronic template installed on all computers in the control practices but containing only the item on current pain intensity.

Individual-level patient outcomes were measured by self-complete postal questionnaires administered to patients shortly after their consultation and at 3-, 6-, and 12-month follow-up and by medical record review. The primary outcome of this trial was pain intensity, measured on a 0–10 scale, with a score of 10 being ‘pain as bad as it can be’. In the primary analysis of the trial this outcome was analysed across 12 months post consultation (i.e. analysis was undertaken across post consultation, 3, 6, and 12 months) using a hierarchical linear mixed model with unstructured covariance, including GP practice (at level 3) and individual participants (at level 2) as random effect variables (a logistic mixed model was used for categorical variables), with repeated measurements of assessment data per individual at level 1. A number of pre-specified covariates were included in the statistical models to help overcome potential selection and confounding bias.

The trial was approved by an independent Research Ethics Committee (11/WM/0093), was prospectively registered (ISRCTN: 40721988), and had a pre-defined protocol, including statistical analysis plan (available from the authors on request). The main findings have been published [13]. The current bias analysis was not included in that pre-specified statistical analysis plan but was instead designed after the primary analysis was completed and the principal trial findings known.

The primary endpoint intention-to-treat analysis found a significantly higher average pain score over the four follow-up time points in the intervention group than the control group (mean difference 0.33, 95% CI 0.05, 0.61; effect size 0.16: 0.02, 0.29). The largest difference of 0.50 was observed at 6-month follow-up. A similar pattern of findings was seen for secondary outcomes.

### Potential for selection bias

In the POST trial, individual participants were identified and recruited *after* randomisation by the treating GP who was not blinded to allocation – a process in which the selective exclusion of ineligible participants was possible. Despite a number of strategies being adopted to mitigate the risks of selection bias, it was noted that a lower proportion of potentially eligible patients were recruited in the intervention arm than in the control arm (16.5% and 21.5%, respectively) and that interviews with GPs in the intervention practices suggested that there might have been ‘selective exclusion of patients at low risk of poor outcome due to perceived irrelevance or intrusiveness of anxiety and depression screening questions in patients with a favourable prognosis or a tendency to reserve screening questions for patients expressing emotional cues/concerns’ [13]. The direction of this selection bias would be capable of producing the observed finding of worse pain outcomes in the intervention arm.

### Bias analysis

For the purposes of this bias analysis we dichotomised the primary pain intensity outcome measure into ‘low pain’ (0–5) and ‘high pain’ (6–10) [14, 15]. Quantitative bias analysis has been developed in, and typically applied to, categorical outcomes. We chose 6-month follow-up as the endpoint of greatest interest as this was when the largest difference between the two arms of the trial was observed in the primary analysis. We also repeated the analysis for 3- and 12-month follow-up time points.

#### Probabilistic bias analysis

To explore the impact of a range of selection probabilities (the probability of being recruited into a trial based on intervention and outcome status) on the treatment effect estimate we undertook probabilistic bias analysis (PBA). This technique requires choosing a distribution from which the samples of the selection odds ratios (ORs) will be drawn. A ‘selection odds ratio’ is calculated from the selection probabilities and used to correct the observed treatment effect OR. We chose the triangular distribution (one of three available and applicable distributions at the time of analysis) as the closest to a normal distribution, and given that there was no evidence to suggest that the data was not normally distributed. The density function for the triangular distribution is given as Equation 1:

1 $$ P(x)=\left\{\frac{2\left(x-a\right)}{\left(b-a\right)\left(c-a\right)}\kern0.9em for\kern0.5em a\kern0.3em \le x\le c,\frac{2\left(b-x\right)}{\left(b-a\right)\left(b-c\right)}\kern0.9em for\kern0.5em c\kern0.3em \le x\le b\right) $$

where *x* ∈ [*a*, *b*] lies between the limits of the distribution, and *c* ∈ [*a*, *b*] is the mode. The triangular distribution is commonly written as:

$$ Triangular\left(a,\kern0.5em b,\kern0.5em c\right) $$

**Fig. 1:**
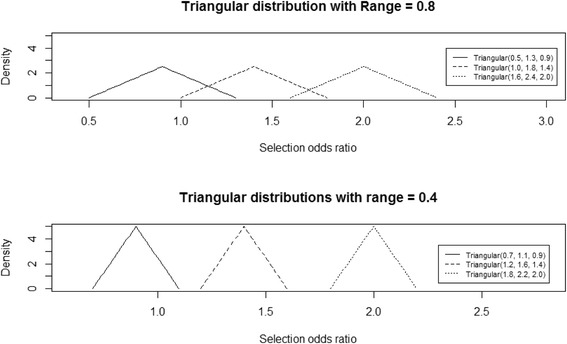
A sample of three triangular distributions sampled from using probabilistic bias analysis (PBA)

We repeated our analyses using a wide (range = 0.8) or narrow (range = 0.4) triangular distribution and each with a mode ranging from 0.9 to 2.0. The distributions thus included examples with greater or lesser uncertainty and that represented more extreme and less extreme (including no) selection bias when compared with the scenario described in the simple bias analysis. Six of the triangular distributions are shown in Fig. 1.

The analyses were applied first to outcome at 6 months and then to outcome at 3 and 12 months.

#### Simple bias analysis

Using methods described by Greenland (1996) and Lash et al. (2009), we undertook a simple bias analysis in which we hypothesised different selection probabilities among potentially eligible patients in intervention and control arms and with respect to outcome status at 6 months. Specifically, we calculated the bias-corrected OR for treatment effect at 6 months under the assumption that the selection probability among potentially eligible patients with ‘high pain’ in the intervention arm was the same as in the control arm. The direction of selection bias in this scenario, therefore, accords with the evidence from qualitative interviews with practitioners. However, it is likely to be extreme, since GPs are imperfect judges of the future pain outcomes of patients [16] and selection of patients perfectly related to outcome is implausible.

All analysis was completed using R studio version 0.99.902 through Windows.

## Results

### Observed results

Table 1 shows the categorised data from the POST trial to be used in the analyses. From the total numbers it can be seen that there was almost double the number of patients available for analysis in the control arm than the intervention for each follow-up time – due to a larger number of allocated practices and larger average size of practices (leading to the higher numbers of potentially eligible patients in the control arm (4238) than the intervention arm (3041)) as well as a higher selection of patients mailed a questionnaire in the control arm (1339 (31.6%) in the control arm versus 703 (23.1%) in the intervention arm).

**Table 1:** New data from the categorised trial outcome for control and intervention arms for each follow-up time point

Follow-up time	3 months		6 months		12 months	
	Intervention	Control	Intervention	Control	Intervention	Control
High pain	162	253	159	236	136	215
Low pain	221	448	215	444	232	429
Total	383	701	374	680	368	644
Potentially eligible before recruitment	3041	4238	3041	4238	3041	4238
Crude OR for treatment effect (95% CI)	1.30 (1.01, 1.67)		1.39 (1.07, 1.80)		1.17 (0.90, 1.53)	

*CI* confidence interval, *OR* odds ratio

At 3 months 383 (12.6%) intervention participants of the 3041 who were initially eligible to participate were available to analyse, compared with 701 (16.5%) in the control arm. This pattern was consistent at the 6- and 12-month follow-up (12.3% and 12.1% for the intervention and 16.0% and 15.2% for the control arm, respectively). Worse pain outcomes were observed in the intervention arm with the observed crude OR (95% CI simulation) at 3, 6, and 12 months: 1.30 (1.01, 1.67), 1.39 (1.07, 1.80), and 1.17 (0.90, 1.53), respectively.

### Probabilistic bias analysis

**Fig. 2:**
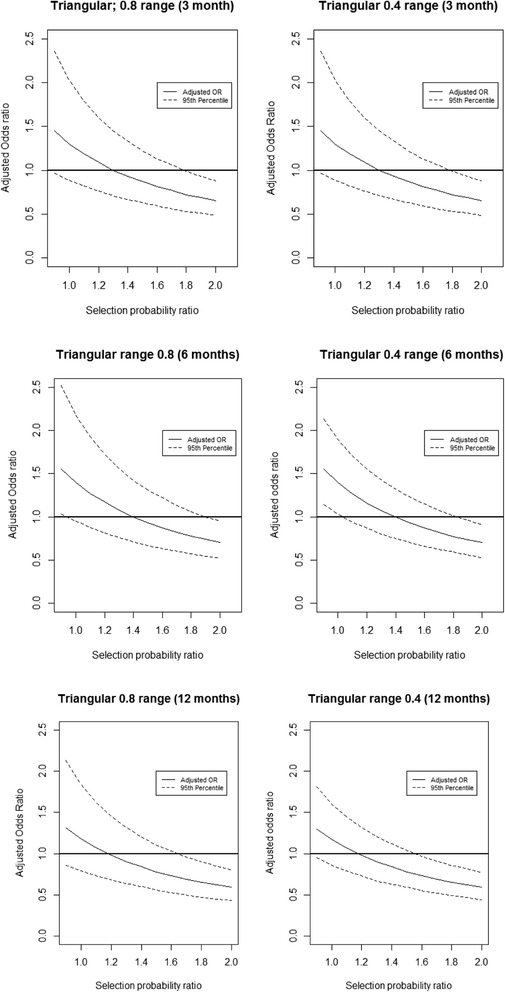
Results of the probabilistic bias analysis (PBA) conducted at each follow-up time point

The results of the probabilistic bias analyses for the outcome at each of the time points and based on a triangular distribution with either a relatively narrow (0.4) or wide (0.8) distribution can be seen in Fig. 2.

As expected, as the selection OR increased above 1, the bias-corrected OR for the pain outcome reduced for all follow-up time points. The finding from simple bias analysis – a bias-corrected OR of 0.91 at 6 months when the selection OR is 1.52 – is now seen in the context of a range of selection ORs. When the selection OR was roughly equal to 1.4 the resulting OR was 1 at 3- and 6-month follow-up, suggesting that the direction of the selection bias that we consider to be highly plausible (i.e. upward of 1 and towards 1.5) would show that there was no difference between intervention and control treatments.

With the larger range of triangular distribution the simulation interval around the adjusted OR was wider, but the overall trend towards 1 was unchanged. This trend appears to begin to level out as it nears an adjusted OR of 0.5; however, the largest value used in this analysis was 2.0.

### Simple bias analysis

As indicated previously, the selection probability for the intervention arm was 0.123 and 0.160 for the control arm at 6-month follow-up. Selection probability combines initial enrolment into the trial and retention to 6 months. If this was nondifferential with respect to the outcome at 6 months, there would be no selection bias and the bias-corrected OR equals the crude OR (Table 2A).

**Table 2:** Simple bias analysis applied to 6-month pain outcome; (A) selection probabilities within treatment arm are assumed to be nondifferential with respect to outcome at 6 months (i.e. no selection bias); (B) selection probabilities within treatment arm are differential with respect to outcome at 6 months, such that the selection probability of patients with high pain is the same in intervention and control arms (i.e. selection bias producing worse outcomes in intervention arm)

(A)	Intervention (=1)	Control (= 0)
Outcome at 6 months = high pain (= A)	n_A1_ = 159	N_A0_ = 236
	*S*_*A1*_ *= 0.123*	*S*_*A0*_ *= 0.160*
	*N*_*A1*_ *= 1293*	*N*_*A0*_ *= 1471*
Outcome at 6 months = low pain (= B)	N_B1_ = 215	n_B0_ = 444
	*S*_*B1*_ *= 0.123*	*S*_*B0*_ *= 0.160*
	*N*_*B1*_ *= 1748*	*N*_*B0*_ *= 2767*
Total potentially eligible	N_1_ = 3041	N_0_ = 4238
Total actually recruited and retained to 6 months	n_1_ = 374	n_0_ = 680
Overall selection probability within treatment arm	S_1_ = 374/3041 = 0.123	S_0_ = 680/4238 = 0.160
Selection odds ratio (OR)	*S*_*A1*_ *S*_*B0*_ */S*_*A0*_ *S*_*B1*_ *= (0.123 × 0.160)/(0.160 × 0.123) = 1*	
Crude $\widehat{OR}$ for treatment effect	n_A1_n_B0_/n_A0_n_B1_ = (159 × 444)/(236 × 215) = 1.39	
(B)	Intervention (= 1)	Control (= 0)
Outcome at 6 months = high pain (= A)	n_A1_ = 159	N_A0_ = 236
	*S*_*A1*_ *= 0.160*	*S*_*A0*_ *= 0.160*
	*N*_*A1*_ *= 991*	*N*_*A0*_ *= 1471*
Outcome at 6 months = low pain (= B)	N_B1_ = 215	n_B0_ = 444
	*S*_*B1*_ *= 0.105*	*S*_*B0*_ *= 0.160*
	*N*_*B1*_ *= 2050*	*N*_*B0*_ *= 2767*
Total potentially eligible	N_1_ = 3041	N_0_ = 4238
Total actually recruited and retained to 6 months	n_1_ = 374	n_0_ = 680
Overall selection probability within treatment arm	S_1_ = 374/3041 = 0.123	S_0_ = 680/4238 = 0.160
Selection OR	*S*_*A1*_ *S*_*B0*_ */S*_*A0*_ *S*_*B1*_ *= (0.160 × 0.160)/(0.160 × 0.105) = 1.52*	
Crude OR for treatment effect	n_A1_n_B0_/n_A0_n_B1_ = (159 × 444)/(236 × 215) = 1.39	
Bias-corrected OR for treatment effect	Crude OR/Selection OR = 1.39/1.52 = 0.91	

Figures in italics in cells are unobserved; *OR* odds ratio; n_ij_ number of participants in that cell, S_ij_ cell selection probability; N_ij_ total participants in each cell if all eligible patients had been recruited

This is contrasted with the bias-corrected OR under the extreme assumption that selection was differential with respect to outcome at 6 months such that selective under-recruitment in the intervention arm affected only those with ‘low pain’ (i.e. a favourable outcome). In this scenario we assumed that the total numbers of patients recruited to the intervention and control arms remained as observed, as did the marginal selection probabilities. The selection probability of the ‘high pain’ group in the intervention arm was fixed at 0.160 (to be equal to control arm). The ‘low pain’ selection probability is, therefore, 0.105. This scenario yields a selection OR of 1.52 and a bias-corrected OR of 0.91.

## Discussion

It is plausible that a selection (bias) OR of 1.2 to 1.4 towards higher-risk patient recruitment to the intervention compared to the control arm occurred within the POST trial. The imbalance in recruitment between the two arms could have introduced selection bias; however, it could also be down to chance. There could have been selection bias present in this study even if the number of patients in each arm had been the same.

To our knowledge the applications of QBA in the existing academic literature are few. The studies that have applied this methodology are cohort or case-control, and involve longitudinal data. In a study investigating the association between smoking and the development of multiple types of cancer, QBA adjusting for misclassification of smoking status was conducted. The results showed that the higher relative risks (RR) were consistently lower than the misclassification-adjusted estimates [17]. Another study investigated misclassification in a case-control setting. They looked into the association dietary patterns on the increase in risk of prostate cancer. After adjustment for misclassification they found slight differences in the ORs in that they were slightly higher than those observed, and that the simulation limits were nearly double the observed [18]. A recent paper involving the use of QBA adjusted for unmeasured confounding. This study looked into the effect an unmeasured confounder had on the association between firearm availability and suicide. Their study showed that the unmeasured confounder would need to be as strong a risk factor as the most potent currently known, such as psychopathology, and socio-economic factors to affect the observed results [19]. However, the authors did go on to suggest that quantifying the extent to which it may change observed results helps to strengthen the results themselves [19].

The results of previous studies, and this report, show the value of conducting a QBA for most kinds of bias present in studies and trials. By adjusting for bias researchers can have more confidence in the results that they are reporting rather than resorting to a theoretical comment in the Discussion section, and it can also help to strengthen the results reported in the study, thereby affecting policies which are built on those reported results [19]. There are other methods for quantifying bias in c-RCTs, notably by using propensity score (PS) methodology when analysing c-RCT data as a cohort [20–22]. Propensity scores are used to adjust the comparison between intervention and control for baseline imbalance in observed and unobserved patient-level characteristics. It is unclear whether QBA is superior to the PS approach. However, approaches that use baseline imbalances could be used to help choose plausible bias scenarios and parameters for QBA.

The main strength of this study is that it is the first that has applied QBA to a c-RCT, thereby providing a framework with which researchers can apply this analysis to c-RCTs where selection bias may be of concern. Another advantage of bias analysis is the flexibility that a researcher has when deciding the direction of the selection bias. In this example we hypothesised, based on observed evidence, that those with low pain/good prognosis were less likely to be recruited into the intervention arm; however, the converse is also plausible, i.e those with high pain were less likely to be recruited to the intervention arm. The bias parameters can be altered to reflect this line of thought.

However, there are a few limitations. The first is that the outcome, pain, had to be transformed into binary for the analysis. This results in a loss of information [23], and potentially an underestimation in the variability of results between the two treatment groups. Secondly, due to the nature of QBA the results could not be adjusted for anything other than the selection bias, hence the resulting ORs are not adjusted for common patient demographics such as age and gender.

Another main limitation to the approach of our evaluation is that no ‘true’ selection probabilities could be calculated as no follow-up questionnaires were sent to those who did not participate. This step, although useful in theoretical platforms, is perhaps less practically implemented in cases such as clinical trials where recruitment is often difficult. The source of the selection bias in this trial can only be hypothetical as voiced by the researchers and it would be impossible to find out if GPs in the control practices were more inclined to recruit different types of patients than those in the intervention due to the nature of the intervention treatment. Another limitation of this study surrounds the formula developed for simple bias analysis. It does not differentiate between data from patients not enrolled in the trial, and those who were lost to follow-up. We were unable to conclude the origin of any selection bias present in this study, whether down to the imbalance in recruitment or to genuine selection bias on the part of the recruiting GPs.

Quantitative bias analysis is a useful tool for insight into the sources of bias present in a trial; however, PBA as used in this study is largely subjective and will be prone to the same counter arguments as that of Bayesian analysis. There are many similarities between PBA and Bayesian methodology, as MacLehose and Gustafson have shown. In broad terms PBA is simply a Bayesian algorithm where the observed data is not updated with each iteration. In both cases one begins with a prior distribution from which iterative samples are taken. The main difference comes from the conditional posterior distribution, where in the Bayesian approach the result depends on the samples taken from the previous iteration of sampling, in PBA the values of the sampled bias parameters are limited to the observed data, hence certain values of bias parameters may be possible in Bayesian platforms, but would be impossible in PBA [24]. However, despite their subtle differences, in some cases PBA can be seen as a Bayesian analysis with a uniform prior [24].

An important area for future research would be to investigate and develop the use of QBA in trial settings, as all existing academic literature has been applied to observational studies. it is acknowledged that in some cases applying QBA to the results of studies results in no difference in the results; however, there have been examples where applying this methodology has provided insights into the effect of bias on the observed results [17, 18].

The POST trial presents a useful case example for illustrating the possible impact that selection bias has on the conclusions of a trial. Using this example we have shown how bias analysis can add to interpretation of results of a c-RCT and planning to routinely obtain empirical data from people excluded from c-RCTs would enhance the utility of bias analysis.

## Conclusion

There was some evidence to suggest that if the assumptions underlying our range of selection bias scenarios were true then selection bias could have affected the results of the POST trial. Rather than being harmful it would appear more likely that there was no difference between the intervention treatment and the control. Evidence of extreme levels of selection of mild patients into the intervention group would be needed to judge that outcomes were actually better in the intervention group than in the control. Applying QBA to a trial provides researchers with more confidence and insight into the conclusions that were produced and should be incorporated into the analysis plan at the design stages of the trial.
